# Exposure to PM2.5 and cardiovascular diseases in Portugal – the contribute of PMCardImpact project

**DOI:** 10.1093/eurpub/ckac131.163

**Published:** 2022-10-25

**Authors:** C Martins, L Lima, S Gonçalves, R Assunção, F Serranheira, S Viegas

**Affiliations:** 1 Occupational and Environmental Health, NOVA National School of Public Health, Universidade NOVA de Lisboa, Lisbon, Portugal; 2 Comprehensive Health Research Center, Lisbon, Portugal; 3 Lisbon School of Health Technology, Polytechnic Institute of Lisbon, Lisbon, Portugal; 4 Centre for Environmental Sciences and Marine Studies, Aveiro, Portugal; 5 IUEM, Instituto Universitário Egas Moniz, Caparica, Portugal

## Abstract

Particulate matter with a diameter of 2.5 μm or less (PM2.5) are one of the air pollutants more detrimental to human health, being responsible for around 400 000 premature deaths in Europe every year. The cardiovascular diseases (CVD) and air pollution are linked, with existing evidence of a causal relationship between exposure to particulate matter and cardiovascular morbidity and mortality. Under the scope of PMCardImpact, a national funded project, data collected from Portuguese air monitoring platform (2005-2021) (>60 stations) was used to estimate the attributable number of cases of acute myocardial infarction. The air monitoring data and parameters such as exposure-response factors will support the risk assessment in AirQ+ software (WHO Regional Office for Europe). Preliminary results showed that exceedances of Air Quality Directive in Portugal ranged between 0.1 % and 10.2% for PM10 and PM2.5 in 2019. Results obtained will include the number of cases of CVD attributable to exposure to PM2.5 in the Portuguese population. Four scenarios of exposure will be considered for presenting the results: current scenario of exposure, new WHO Air Quality guidelines, European Commission Air Quality Directive and lastly, a worst-case scenario. This assessment will be the starting point for calculation of the burden of disease of CVD that exposure to PM2.5 represent in Portugal. With a view to promote the science to policy interface, PMCardImpact project will make available to policy makers the needed supporting information to act, including actionable knowledge on air pollution trends and related health effects, to implement reducing air pollution policies.

This work is funded by FCT/MCTES through national funds to PMCardImpact (EXPL/SAU-PUB/0944/2021) and CESAM (UIDP/50017/2020 + UIDB/50017/2020 + LA/P/0094/2020).

**Key messages:**

• PMCardImpact will make available to policy makers the needed supporting information to act to implement reducing air pollution policies.

• Risk assessment will allow to determine the number of CVD cases attributable to air pollution.

